# DiSMix: Dimensional Swap Mix for Feature-Level Data Augmentation in Vision Transformers

**DOI:** 10.3390/jimaging12060223

**Published:** 2026-05-25

**Authors:** Rinka Kiriyama, Akio Sashima, Ikuko Shimizu

**Affiliations:** 1 Department of Electrical Engineering and Computer Science, Tokyo University of Agriculture and Technology, 2-24-16 Naka-cho, Koganei, Tokyo 162-0061, Japan; kiriyama@m2.tuat.ac.jp (R.K.); sashima-akio@aist.go.jp (A.S.); 2 Research Institute on Human and Societal Augmentation, National Institute of Advanced Industrial Science and Technology, Kashiwa II Campus, University of Tokyo, 6-2-3 Kashiwanoha, Kashiwa, Chiba 277-0882, Japan

**Keywords:** vision transformer, data augmentation, mixup, dimensional swap mix (DiSMix)

## Abstract

Mixup is a data augmentation technique that improves prediction accuracy in classification tasks by combining representations of training samples, which makes it particularly effective in settings with limited data and during fine-tuning for downstream tasks. However, representations generated by mixup may appear unnatural, which can negatively affect fine-tuning performance. To address this limitation, we propose a vision transformer (ViT)-aware variant of mixup strategies, Dimensional Swap Mix (DiSMix). DiSMix divides a representation vector into two segments corresponding to subspaces of the original feature space and generates new representations by swapping one segment with that from another sample and concatenating the segments. This allows part of the original representation to remain unchanged, enabling the model to learn from partially preserved features. We evaluate DiSMix by applying several mixup-based methods to fine-tune ViTs on the VTAB-1k benchmark. The findings show that DiSMix improves accuracy on the VTAB-1k Natural split, reaching 80.0%, compared with conventional mixup methods. This suggests that DiSMix is an effective alternative for representation-level data augmentation in fine-tuning scenarios.

## 1. Introduction

In recent years, transformer-based architectures have become the foundation of interactive and generative AI [1,2], and they are widely used across various applications. In computer vision, a vision transformer (ViT) is used to recognize semantic meanings of images in various tasks such as image classification [3,4], action recognition [5,6] and visual question answering [7].

To improve accuracy and generalization, data augmentation is commonly applied during training and fine-tuning. Conventional augmentation techniques include color jittering, pixel shifting, flipping, and mixup [8].

**Figure 1:**
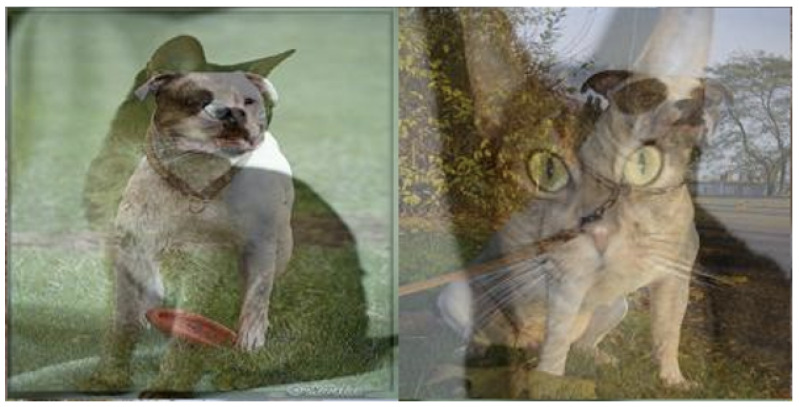
An example of images generated using mixup. This is a result of mixup using two images in the category “Pets”.

Mixup generates synthetic training samples by linearly interpolating between two examples, and improves robustness and generalization by promoting linear behavior between classes (Figure 1). Since then, several variants have been proposed, including manifold mixup [9] and MSMix [10], thus expanding mixup-style augmentation beyond the input space. However, these methods may generate unnatural representations, particularly when data are limited, which can confuse the model and degrade fine-tuning performance on downstream tasks. In particular, since it is difficult to track the contribution of individual feature dimensions to the final prediction, these methods may inadvertently promote overfitting to specific feature dimensions.

This issue is especially pronounced in ViTs. Unlike convolutional models, ViT representations consist of multiple spatial tokens that share correlated feature dimensions due to linear patch embeddings, making it unlikely that previous feature-swapping methods replace a given dimension across all tokens simultaneously. The model can therefore continue to rely on such dimensions in subsequent processing. Furthermore, self-attention in ViTs propagates spatial relationships across all tokens. As a result, local modifications introduced by previous methods may be absorbed into the global representation, weakening the regularization effect of mixup and leaving open the possibility of overfitting to specific feature dimensions. As a result, feature-level mixup strategies originally designed for other modalities, such as MSMix in Natural Language Processing, may not effectively constrain ViT representations.

To address this limitation, we propose Dimensional Swap Mix (DiSMix), a ViT-aware variant of feature-level augmentation strategies. Rather than interpolating feature values, DiSMix swaps feature dimensions between samples in a dimension-consistent manner. A single binary decision is made for each feature dimension and shared across all tokens, ensuring that each dimension is consistently derived from a single source throughout the representation. This design enables the model to avoid over-reliance on specific feature dimensions. Unlike input-level mixup, this approach also avoids generating unnatural pixel-level interpolations. The swapping operation is applied before transformer blocks, where feature dimensions retain semantic correspondence to linear projection filters, making dimensional constraint both meaningful and effective.

We evaluate DiSMix on the VTAB-1k benchmark using fine-tuned ViT models and compare it with several mixup-based methods. DiSMix achieves strong performance across tasks, outperforming conventional mixup variants on VTAB-1k Natural tasks and reaching 80.0% accuracy. We further introduce a manifold variant, MDiSMix, which applies dimension-consistent swapping within hidden layers and demonstrates superior performance on VTAB-1k Structured tasks. Beyond performance gains, our analysis suggests that DiSMix may promote a flattening tendency in representations by constraining high-variance feature directions, which is qualitatively consistent with the theoretical motivation of manifold mixup while avoiding pixel-level interpolation. We emphasize that this interpretation is qualitative and reflects observed tendencies rather than a verified theoretical property.

The following are the main contributions of the present study:

- We introduce a dimension-consistent feature swapping scheme designed for ViT architectures to discourage overfitting to specific feature dimensions caused by spatial attention propagation.
- We propose a pre-transformer mixing strategy that leverages the dimension–filter correspondence in ViTs, preserving semantic structure without unnatural interpolation.
- We demonstrate strong downstream performance on VTAB-1k, where DiSMix and its manifold variant achieve competitive or superior accuracy across task categories.
- We provide empirical evidence of representation flattening induced by dimensional constraints, offering insight into how controlled feature swapping can improve generalization in fine-tuning scenarios.

## 2. Related Works

### 2.1. Mixup

Data augmentation is used to enhance the training datasets. Among various data augmentation methods, mixup-based ones have been introduced to expand the diversity of the models. The typical mixup-based method [8] generates new training examples by linearly combining existing training data sets. For the given two examples, $\left(x_{i},y_{i}\right)$ and $\left(x_{j},y_{j}\right)$, from the training dataset, mixup generates a new example $\left(\tilde{x},\tilde{y}\right)$ according to the following equations:

(1) $$\begin{array}{cc} \tilde{x} & =\lambda x_{i}+\left(1-\lambda \right)x_{j}, \end{array}$$

(2) $$\begin{array}{cc} \tilde{y} & =\lambda y_{i}+\left(1-\lambda \right)y_{j}, \end{array}$$

where λ∼Beta(α,α), and α is the hyperparameter. Mixup has been shown to improve generalization performance and enhance robustness against small changes in representation, known as adversarial attacks, thereby increasing recognition stability [11].

Many methods that follow the original mixup method are typically categorized into two types:

- Methods that modify part of the image area to produce intermediate representations of different classes (cutting-based);
- Methods that mix hidden representations to generate intermediate features (mixing-based).

Note that these methods may also produce unnatural and ambiguous representations, which negatively affect the models [12].

#### 2.1.1. Cutting-Based Mixup

Cutting-based methods replace certain areas of the input image with patches from other images. For instance, CutMix [12] proposes randomly cutting out a part of an image and replacing it with a region from another image. This technique is commonly used during training for class recognition tasks, such as those involving ImageNet-1K [13].

Unlike mixup, CutMix does not generate ambiguous or unnatural expressions because the original representation of local regions remains unchanged. However, since this method leads to the loss of part of the image, information crucial for class detection may be removed. To address this issue, some methods have proposed more careful area selection. For example, TokenMix [14] detects areas based on the class activation map. This approach seems to work well when there is sufficient knowledge about the image set. However, it may be less effective when applied to downstream tasks like VTAB-1k [15], where such prior knowledge may be limited. In particular, linear interpolation may cause the model to fit blended pixel-level patterns that do not exist in the true data distribution, which can weaken the model’s ability to recognize original, unmixed images in downstream tasks.

#### 2.1.2. Mixing-Based Mixup

Mixing-based methods combine feature values in a manner similar to the original mixup. Among these, manifold-based methods apply mixup to hidden representations at a specific layer *k*. Suppose we are training a deep neural network defined as $f\left(x\right)=f_{k}\left(g_{k}\left(x\right)\right)$. Manifold mixup [9] generates the mixed representation $\tilde{x}$ from g(x) as follows:

(3) $$\tilde{x}=\lambda g_{k}\left(x_{i}\right)+\left(1-\lambda \right)g_{k}\left(x_{j}\right).$$

While manifold mixup focuses on reducing the variance of class representations, other mixup-based approaches emphasize class-specific representations. For example, SwapMix [16] exchanges object features encoded by an RCNN with those of other objects in vision question answering tasks.

Another approach which focuses on the hidden representation is MSMix [10] in Natural Language Processing, which exchanges features in the hidden representation. Formally, MSMix is expressed by the following equation:

(4) $$\hat{h}=M_{\mathrm{MS}}⊙h_{1}+\left(1-M_{\mathrm{MS}}\right)⊙h_{2},$$

where $M\in {\left\{0,1\right\}}^{L\times D}$, *L* is the length of tokens, *D* is the dimensions of each token, and *h* is the hidden representation. MSMix indicates the exchanged features by setting the value of L×⌊λD⌋ in *M* to 1 and the rest to 0. Note that MSMix is equivalent to applying manifold mixup with a binary mask in the feature space. Based on this approach, we propose the feature swapping method, which is suited for image processing.

However, MSMix—originally designed for NLP—replaces features independently across token positions, making it unsuitable for ViTs, where self-attention propagates information globally across all tokens. This replacement may fail to resolve overfitting to specific feature dimensions, as local modifications introduced at individual token positions may be absorbed by the global representation through attention interactions.

## 3. Method: Dimensional Swap Mix (DiSMix)

### 3.1. Overview and Design Principle

In this section, we describe DiSMix, a dimension-consistent feature swapping method designed for ViTs. The core principle of DiSMix is to enforce a single binary decision per feature dimension shared across all tokens, so that swapped dimensions are less likely to be reconstructed via spatial redundancy. This design specifically addresses the structural redundancy of ViTs, where spatial tokens share correlated feature dimensions.

We first formalize the problem setting and notation, then introduce the dimension-consistent masking scheme, and finally discuss where and how the swapping operation is applied during training.

### 3.2. Problem Setting and Notation

Let f(·) be a ViT fine-tuned for a downstream classification task. Given two training samples, $\left(x_{i},y_{i}\right)$ and $\left(x_{j},y_{j}\right)$, we denote the token-level feature representations extracted at a pre-transformer stage (i.e., after patch embedding and positional addition) by

$$X_{1}=\phi \left(x_{i}\right)\in R^{L\times D},\;X_{2}=\phi \left(x_{j}\right)\in R^{L\times D},$$

where *L* is the token length and *D* is the feature dimension per token.

In ViTs, each feature dimension corresponds to a shared linear projection filter that is applied uniformly across all tokens. As a result, different spatial tokens often exhibit correlated variations along the same feature dimensions. This property motivates enforcing dimension-wise consistency during mixing, rather than performing independent replacements at the token level.

Our objective is to construct a mixed representation $\hat{X}$ that (i) avoids pixel-level interpolation artifacts and (ii) reduces potential information leakage caused by spatial redundancy in ViT representations.

### 3.3. Dimension-Consistent Binary Masking

DiSMix generates a *binary, dimension-wise* mask that is shared across the *L* tokens:

$$M_{\mathrm{DiS}}=\mathrm{diag}\left(b_{1},b_{2},\ldots ,b_{D}\right)\in {\left\{0,1\right\}}^{D\times D},\;b_{d}\in \left\{0,1\right\}.$$

The mixed representation is defined as

(5) $$\hat{X}=X_{1}M_{\mathrm{DiS}}^{⊤}+X_{2}{\left(I-M_{\mathrm{DiS}}\right)}^{⊤},$$

which replaces the *same* subset of feature dimensions across all tokens. In contrast to token-wise replacement, Equation (5) reduces the likelihood that tokens retain alternative sources for a swapped dimension, thereby strengthening the intended regularization. Unlike MSMix, which applies independent masking at each token position, DiSMix applies a single mask across all tokens, enforcing a global dimensional constraint.

### 3.4. Sampling the Number of Swapped Dimensions

To control the expected swapping ratio, we first sample a continuous mixing coefficient λ∼Beta(α,α) and define the number of dimensions to be preserved from $X_{1}$ as k=⌊λD⌋. We then define the binary entries using a deterministic assignment,

$$b_{d}=\left\{\begin{array}{cc} 1, & \mathrm{if}\;d\in S,\;|S|=k,\\ 0, & \mathrm{otherwise}, \end{array}\right.$$

where S is a uniformly random subset of {1,…,D} of size *k*. This two-step procedure (continuous λ→ binary mask) controls the expected swap ratio while avoiding ambiguous interpolation.

### 3.5. Where to Mix: Pre-Transformer Swapping

DiSMix applies the dimension-consistent swapping operation before the transformer blocks, directly on the patch embedding outputs. At this stage, feature dimensions maintain a clear semantic correspondence to linear projection filters, making dimension-wise constraints both interpretable and effective. Applying swapping after multiple self-attention layers may entangle feature dimensions through attention aggregation, weakening the intended regularization and obscuring the interpretability of swapped dimensions. Pre-transformer swapping therefore provides a principled balance between semantic alignment and effective regularization.

### 3.6. Training Objective

Following mixup-style supervision, the target label is linearly combined:

$$\hat{y}=\lambda y_{i}+\left(1-\lambda \right)y_{j},\;\lambda ∼\mathrm{Beta}\left(\alpha ,\alpha \right),$$

Then, the model is trained to minimize the cross-entropy $L_{\mathrm{CE}}\left(f\left(\hat{X}\right),\hat{y}\right)$. Note that only the *labels* are interpolated; the *features* are *not* interpolated but are *swapped* along selected dimensions (Equation (5)). This avoids synthesizing out-of-distribution pixel patterns while still encouraging linear behavior in the label space.

### 3.7. Relation to Prior Mixing Strategies

Existing mixup-based methods can be broadly categorized as follows:

- Pixel- or patch-level mixing (e.g., mixup, CutMix) perturbs the input image space and may produce visually unnatural samples, particularly in low-data regimes.
- Token-wise feature replacement (e.g., MSMix-like strategies) swaps features independently across tokens, which may fail to discourage overfitting to specific feature dimensions, as local modifications may be absorbed by the global representation through attention interactions.

DiSMix addresses both limitations by enforcing dimension-consistent swapping without pixel-level interpolation. By constraining entire feature dimensions across all tokens, DiSMix explicitly limits the number of high-variance feature directions available for recognition. This constraint may encourage the model to rely on more stable and distributed features, thereby improving generalization during fine-tuning.

### 3.8. Optional Manifold Variant (MDiSMix)

We further introduce a manifold variant, MDiSMix, which applies the same dimension-consistent swapping at an intermediate hidden layer $g_{k}\left(\cdot \right)$, where *k* is sampled uniformly at random from all transformer blocks at each training step:

$${\hat{X}}^{\left(k\right)}=H_{1}^{\left(k\right)}M_{\mathrm{DiS}}^{⊤}+H_{2}^{\left(k\right)}{\left(I-M_{\mathrm{DiS}}\right)}^{⊤},\;H_{\ell }^{\left(k\right)}=g_{k}\;\left(\phi (x_{\ell })\right),$$

This is followed by $f_{k}\left({\hat{X}}^{\left(k\right)}\right)$ for prediction. This variant may encourage representation flattening at deeper layers and is particularly effective for structured downstream tasks, where abstract relational reasoning benefits from stronger regularization. Our experiments demonstrate that DiSMix and MDiSMix exhibit complementary strengths across task categories.

This variant is optional, as non-manifold DiSMix is more effective for natural image tasks, while MDiSMix is particularly beneficial for structured tasks.

### 3.9. Comparison with Related Feature-Level Mixing Methods

To clarify the conceptual position of DiSMix, we summarize the key differences between DiSMix and representative mixup-based augmentation methods in Table 1. Although these methods share the common objective of improving generalization through feature mixing, they differ substantially in what is mixed and how the mixing operation is constrained.

Conventional mixup and CutMix operate in the input space by interpolating pixels or replacing image regions, which can introduce visually ambiguous samples and, in some cases, out-of-distribution artifacts. Feature-level approaches such as manifold mixup extend value interpolation to hidden representations, encouraging linear behavior in the representation space but still allowing mixed features to be reconstructed from redundant sources.

Token-wise feature replacement methods, including MSMix-style strategies, further avoid interpolation by exchanging subsets of features at individual token positions. However, when applied to ViTs, this token-level independence may be insufficient to prevent overfitting to specific feature dimensions. Since self-attention propagates information globally across all tokens, local modifications introduced at individual token positions may be absorbed by the global representation, leaving dimension-wise overfitting unresolved.

In contrast, DiSMix enforces dimension-consistent swapping, where a single binary decision is applied per feature dimension and shared across all tokens. This design explicitly prevents alternative information sources for swapped dimensions and aligns with the structural properties of ViT representations. Rather than interpolating values or relying on token-wise redundancy, DiSMix constrains entire feature dimensions, encouraging the model to rely on remaining distributed cues during fine-tuning.

This perspective highlights that DiSMix can be viewed not only as a variant of existing mixup methods, but as a structurally motivated augmentation strategy tailored to vision transformers, addressing limitations that arise when feature-level mixing methods are directly transferred from other domains.

## 4. Results

In this paper, we focus on mixing-based mixup and compare the accuracy for six mixup methods. For MSMix and DiSMix, we denoted the non-manifold approach as SMix and the manifold approaches as DiSMix and MDiSMix. In addition, we refer to the method of not using any mixup as ERM.

### 4.1. Experimental Setting

Fine-tuning was performed on a set of downstream tasks called VTAB-1K [15] using the pretrained weights of Vanilla-ViT [3]. (Details of VTAB-1k can be found in Appendix A.) The experimental parameters were set as shown in Table 2, based on the results of Jia et al. [17]. At the end of each epoch, we evaluated the model’s performance using the validation dataset, keeping track of the model with the highest validation score (or lowest validation loss in case of ties). After completing all training epochs, we used this best-performing model to evaluate the accuracy on the test dataset, which we report as our final performance metric.

To determine the most suitable learning rate, we performed a grid search for the range $\left\{6.3\times 10^{-6},\;1.3\times 10^{-5},\;2.5\times 10^{-5},\;5.0\times 10^{-5}\right\}$ for VTAB-1K Natural and VTAB-1K Specialized tasks. However, we found that this range might not contain the optimal learning rate for VTAB-1K Structured tasks. Therefore, we conducted a supplementary experiment to determine the base learning rate for these tasks, which is described in Appendix B. Then, we performed a grid search around this base learning rate using four values: one-quarter of the base rate, one-half of the base rate, the base rate itself, and twice the base rate. The optimal learning rate was selected via grid search. The final test accuracy is reported as the mean ± standard deviation over ten seeds {20, 42, 55, 59, 74, 98, 131, 173, 224, 264}.

### 4.2. Main Results

Table 3 presents the results of fine-tuning a pre-trained ViT-B/16 model across VTAB-1k subtasks. The values represent Top-1 accuracy, with the change in accuracy compared to the Empirical Risk Minimization (ERM) baseline shown in parentheses.

Our proposed methods, DiSMix and MDiSMix, demonstrate competitive performance across different task categories. Notably, DiSMix outperforms other mixup methods on VTAB-1K Natural tasks, achieving the highest accuracy of 80.0% (a 1.7% improvement over ERM). MDiSMix exhibits superior performance on VTAB-1k Structured tasks, with an accuracy of 59.6% (a 3.6% improvement over ERM). Moreover, MDiSMix achieves the best overall performance across all VTAB-1k tasks, with a total accuracy of 72.0% (a 1.5% improvement over ERM).

### 4.3. VTAB-1k Natural

Table 4 and Table 5 present the results for the VTAB-1k Natural tasks. Our proposed DiSMix method demonstrates superior performance, outperforming other methods in five out of seven tasks. Notably, DiSMix achieves higher accuracy than other methods across all VTAB-1K Natural tasks, with the exception of fine-grained classification tasks (Flowers and Pets).

It is worth noting that fine-grained classification tasks typically require distinctions based on more specific features. For instance, species detection may necessitate the identification of not only body hair but also its color or length. DiSMix employs a method where some dimensions are randomly swapped, effectively forcing the model to evaluate classes with restricted dimensions. This approach may limit the model’s access to highly important features during training, potentially lacking information in fine-grained classification tasks.

The performance discrepancy observed in fine-grained tasks (Flowers and Pets) suggests that while DiSMix’s dimensional swapping strategy is beneficial for most Natural tasks, it may not be optimal for tasks requiring very detailed feature analysis. This observation highlights the trade-off between generalization and fine-grained discrimination in data augmentation techniques, and suggests potential areas for further investigation and improvement in the DiSMix method.

### 4.4. Generalization to Other Architectures

To assess the generalizability of DiSMix, we evaluated it on VTAB-1k Natural tasks using Swin transformer V2 (SwinV2) [18] as an alternative backbone. Table 6 and Table 7 present the results (accuracy is reported over five seeds {20, 42, 55, 59, 74}). DiSMix achieved the highest average accuracy, outperforming other augmentations. This is consistent with the results obtained using ViT-B/16, suggesting that the dimension-consistent swapping strategy is effective across different ViT architectures.

### 4.5. The Effects of Manifold Mixup

**Figure 2:**
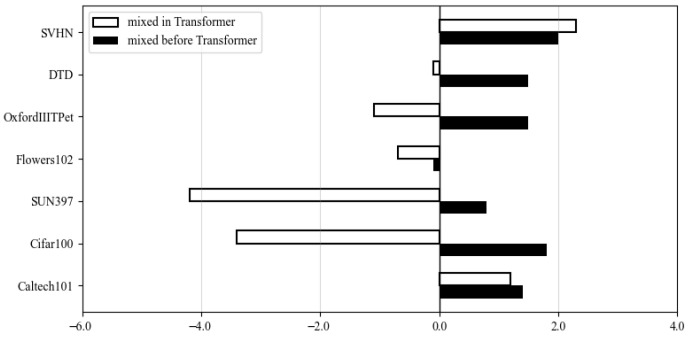
A comparison of increasing rates between manifold and non-manifold approaches in VTAB-1k Natural.

**Figure 3:**
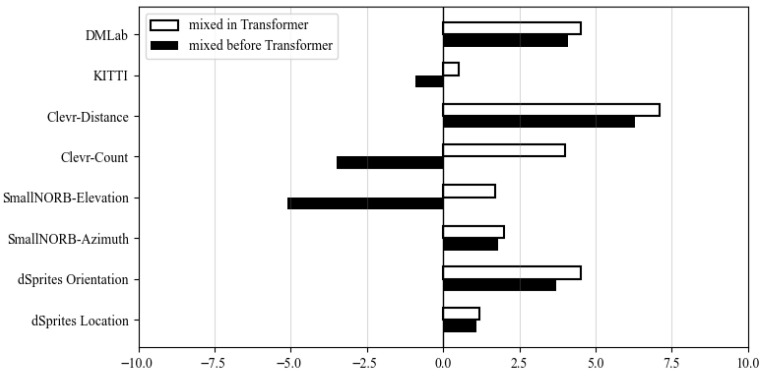
A comparison of increasing rates between manifold and non-manifold approaches in VTAB-1k Structured.

As demonstrated in Section 4.2, DiSMix outperforms other methods on most tasks in VTAB-1k Natural, while MDiSMix shows superior performance in VTAB-1k Structured tasks. Our experiments also revealed that the effects of manifold mixup are primarily observed in VTAB-1k Structured tasks. Figure 2 and Figure 3 visualize the improvement in accuracy for VTAB-1k Natural and VTAB-1k Structured tasks, respectively, comparing methods with and without the application of manifold mixup against the ERM baseline.

The results reveal an interesting pattern: for VTAB-1k Natural tasks, non-manifold methods often yield improved accuracy compared to ERM, while manifold methods sometimes lead to reduced accuracy. Conversely, for VTAB-1k Structured tasks, the opposite trend is observed, with manifold methods generally showing better performance. This difference in performance suggests that the effectiveness of manifold mixup is task-dependent. The method appears to be particularly beneficial for structured tasks, which often involve more complex relationships between features. On the other hand, natural tasks, which typically rely on more direct visual features, seem to benefit more from non-manifold approaches like DiSMix. These findings highlight the importance of selecting appropriate data augmentation techniques based on the specific characteristics of the task at hand. They also underscore the potential for developing adaptive mixup strategies that can leverage the strengths of both manifold and non-manifold approaches depending on the task type.

## 5. Discussion

### 5.1. The Difference Between Mixup and DiSMix

In the previous section, we observed that mixup exhibits certain expressive unnaturalness. Here, we discuss the implications of learning with mixup, based on our experimental results. We focus on the variable α, which is integral in determining the mixing ratio λ. Table 8 and Table 9 demonstrate the accuracy improvements when changing the condition from α=1.0 to α=0.5, showing that this difference is less pronounced for DiSMix compared to mixup, except for the SUN397 and Flowers datasets.

When α decreases from 1.0 to 0.5, λ is more likely to be close to 0 or 1, resulting in generated images resembling one of the original images. Conversely, α=1.0 produces more balanced combinations. These mixing behaviors present different learning challenges: with α=0.5, the model learns from images often resembling originals, while α=1.0 requires discerning features from evenly blended images.

The examples generated by mixup often contain unnatural expressions, potentially leading the model to treat each representation independently, without recognizing the relationship to the expressions before applying mixup. In contrast, DiSMix generates examples by combining two representation vectors, each corresponding to a subset of the distribution that produces the original representation. This approach preserves more of the underlying structure of the original data distributions, creating mixed samples that retain interpretable relationships to the original classes.

**Figure 4:**
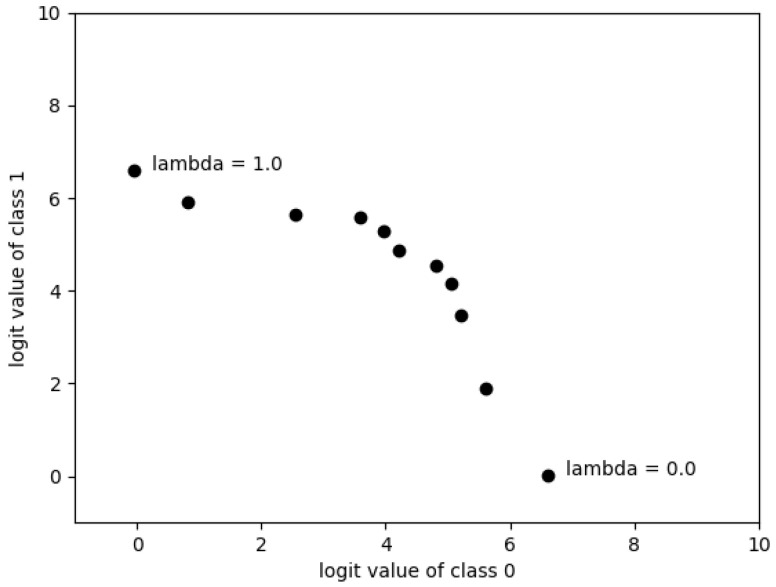
Visualization of the prediction of mixed images generated by mixup.

**Figure 5:**
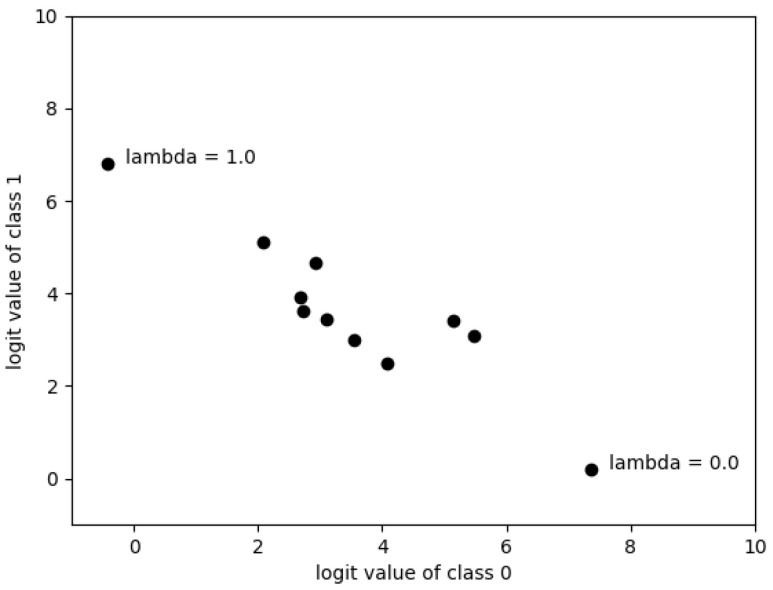
Visualization of the prediction of mixed images generated by DiSMix.

To investigate the possibility of unnatural representations in more detail, we visualize the prediction results when mixup is applied to the learned model. Figure 4 and Figure 5 show the results for mixup representations of two classes from the Pets dataset, plotted according to the model’s predicted logit values. In these plots, class 0 is represented by a horizontal axis, and class 1 by a vertical axis. Each plot corresponds to the average of logits for certain λ values.

In the mixup results, the sequence of points tends to be convex at λ=0.5, suggesting appropriate prediction for mixed expressions around this point. These results suggest that while mixup learning excels at recognizing mixed expressions, it struggles to effectively apply this knowledge to improve inference on original, unmixed images. That is to say, mixup may not fully bridge the gap between mixed and original representations.

The difference between mixup and DiSMix underscores the importance of maintaining meaningful structure when generating mixed samples for data augmentation. By not only introducing diversity through mixing but also preserving interpretable links to the original data distributions, DiSMix is considered to achieve the superior prediction scores described earlier.

### 5.2. The Difference Between SMix (MSMix) and DiSMix

**Figure 6:**
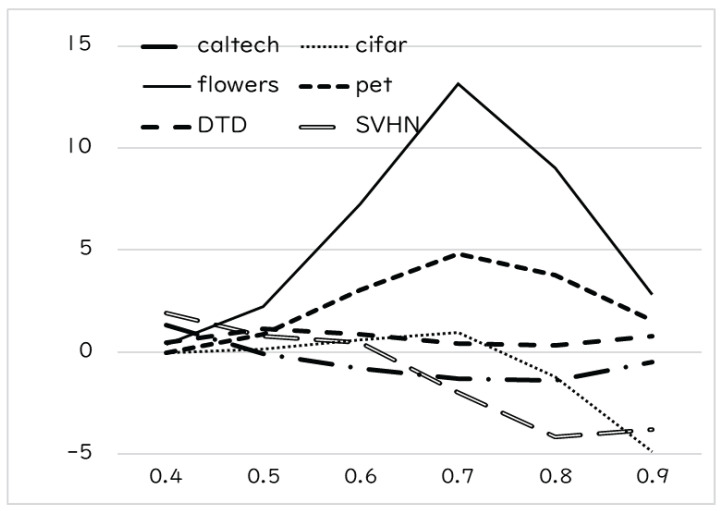
Comparison of SMix and DiSMix performance when exchanging a portion of features with noise. The vertical axis shows the accuracy difference between DiSMix and SMix, with positive values indicating higher accuracy for DiSMix. λ represents the amount of change, where larger values correspond to a lower percentage of noise.

To investigate the differences between SMix and DiSMix, we compared their accuracy when images and noise are mixed. Figure 6 presents the comparison results of SMix and DiSMix when a portion of features are exchanged with noise. The following key observations were made across different datasets:

- In the CIFAR and SVHN datasets, SMix outperformed DiSMix in accuracy when the ratio of image feature dimensions was 0.9 (noise area was 0.1).
- In the Flowers and Pet datasets, DiSMix significantly surpassed SMix in accuracy when the ratio of image feature dimensions was 0.7.

These observations underscore the advantages and limitations of DiSMix compared to SMix. In DiSMix-based learning, the loss of features in specific token dimensions forces the model to make inferences based on limited information. This compels the model to perform class distinctions using effectively low-rank vector information, as specific dimensions common to all tokens are lost.

SMix may outperform DiSMix when tokens exhibit redundancy, as it can compensate for dimension loss by utilizing features from similar tokens. As shown in Figure 6, SMix’s accuracy surpasses that of DiSMix for low-resolution images like CIFAR and SVHN, especially with small noise ratios. This is due to lower-resolution images having fewer representation patterns and more similar tokens. In near-perfect conditions, SMix can complete missing information, while DiSMix cannot due to complete feature loss in certain dimensions.

Conversely, DiSMix outperforms SMix in fine-grained classification tasks when the noise ratio is 0.7. This suggests DiSMix enables broader classifications even when detailed subdivisions are not feasible due to features in certain dimensions being replaced with noise. This advantage stems from the unification of exchanged dimensions across tokens. DiSMix prevents the model from searching entire tokens for important features and increases the representation power of token sub-dimensions by dropping information in certain dimensions. This approach clarifies each vector dimension’s role, enabling predictions via sub-dimensions analogous to weak learners in ensemble learning.

In conclusion, DiSMix’s complete loss of features in certain dimensions results in each remaining dimension carrying more significance compared with SMix. SMix can access information from other tokens even after feature swapping, which sometimes works well in image-processing tasks with token redundancy. Conversely, DiSMix exhibits greater expressive power with limited dimensions; however, it struggles in situations that require higher dimensions. These characteristics define the distinct strengths of each method.

### 5.3. Limitations

Despite its effectiveness across a wide range of downstream tasks, the proposed DiSMix framework has several limitations that warrant further investigation.

First, DiSMix may be less suitable for fine-grained visual recognition tasks that require highly detailed and localized feature distinctions, such as species-level classification in the Flowers or Pets dataset. By design, DiSMix enforces complete removal of selected feature dimensions across all tokens, which can disproportionately suppress dimensions that encode subtle but critical attributes. While this dimensional constraint improves generalization in many settings, it may reduce discriminative capacity when fine-grained cues are essential. This trade-off suggests that DiSMix favors robustness and feature redistribution over maximal expressiveness.

Second, the current implementation of DiSMix employs uniform random selection of feature dimensions for swapping, without considering the relative importance or semantic role of individual dimensions. In ViTs, certain feature dimensions may be more strongly associated with specific visual concepts or task-relevant signals. Ignoring such differences may lead to suboptimal feature suppression, particularly for tasks with skewed feature importance distributions. Incorporating adaptive or importance-aware dimension selection strategies could be a promising direction for improving performance consistency across task types.

Third, DiSMix is primarily designed around the standard ViT architecture and its linear patch embedding mechanism, which ensures semantic alignment across feature dimensions. Its effectiveness under alternative architectures—such as hybrid CNN–transformer models, hierarchical ViTs, or models with dynamic token pruning—has not yet been explored. In such architectures, the assumption of globally shared and semantically consistent feature dimensions may be weakened, potentially reducing the benefit of dimension-consistent swapping.

Fourth, while we demonstrated that DiSMix and its manifold variant exhibit task-dependent strengths on VTAB-1k, the current framework does not adaptively select between pre-transformer and manifold-level swapping during training. An automated or task-aware strategy that dynamically adjusts the mixing location or intensity could further improve performance and usability in practical fine-tuning scenarios.

Finally, the current formulation of DiSMix adopts soft label interpolation following the convention of existing mixup-based methods. However, since DiSMix performs discrete feature swapping rather than continuous feature interpolation, the appropriateness of linear label blending is not theoretically guaranteed. A more principled label assignment strategy, such as one based on knowledge distillation and contrastive learning, could better reflect the semantic content of the augmented representations.

## 6. Conclusions

In this study, we explored feature-level mixup strategies for fine-tuning ViTs in low-data regimes and proposed DiSMix, a dimension-consistent feature swapping method. DiSMix replaces entire feature dimensions across all tokens using a shared binary mask, rather than interpolating values or performing token-wise feature replacement. This design is motivated by the structural properties of ViT representations, where feature dimensions are shared across spatial tokens through linear patch embeddings. The experimental results on the VTAB-1k benchmark indicate that DiSMix achieves competitive performance relative to existing mixup-based methods, with consistent improvements observed on several natural image recognition tasks. By partially preserving original representations while constraining selected feature dimensions, DiSMix provides an alternative way to regularize ViT representations during fine-tuning without introducing pixel-level interpolation artifacts. We also examine a manifold variant, MDiSMix, which applies the same dimension-consistent swapping strategy to intermediate hidden layers, and show that this approach can be advantageous for structured tasks that require learning relational or abstract feature dependencies. Our analysis suggests that enforcing dimensional consistency across tokens can influence representation geometry by limiting the dominance of high-variance feature directions, offering a possible explanation for the observed performance trends. The findings also highlight that the effectiveness of feature-level mixing strategies is task-dependent, and that no single mixing scheme uniformly outperforms others across all task categories. Overall, this study shows that dimension-aware feature swapping is a viable and principled augmentation strategy for ViT fine-tuning, particularly when data availability is limited. While the scope of our experiments is restricted to standard ViT architectures and VTAB-1k tasks, we hope that the insights presented here will inform future work on adaptive and architecture-aware mixup strategies for transformer-based vision models.

## Figures and Tables

**Table 1:** Conceptual comparison between DiSMix and representative mixup-based augmentation methods. × for not good, ∆ for partially ok, and ✓ for ok.

Method	Mixing Level	Mixing Unit	Dimensional Consistency	ViT-Aware Design
Mixup [8]	Input	Pixel values	×	×
CutMix [12]	Input	Image regions	×	×
Manifold mixup [9]	Hidden	Feature values	×	×
MSMix/SMix [10]	Hidden	Token-wise features	×	∆
DiSMix (ours)	Hidden	Feature dimensions	✓	✓

**Table 2:** Learning settings for fine-tuning.

Optimizer	AdamW
Weight decay	0.0
Learning rate schedule	cosine
Mixup alpha	{0.5,1.0}
Warmup epoch	5
Learning epoch	100
Crop method	Random (training)/Center (testing)
Crop percentage	0.95

**Table 3:** Results of fine-tuning on VTAB-1k tasks. Text in blue indicates the improvement relative to ERM, while text in red indicates the deterioration relative to ERM.

	VTAB-1k			
Method	Natural	Specialized	Structured	Total
ERM	78.3	85.8	56.0	70.5
Mixup	79.3 (1.0)	86.2 (0.4)	56.2 (0.2)	71.0 (0.5)
Manifold	77.1 (−1.2)	86.1 (0.3)	58.6 (2.6)	71.2 (0.7)
SMix	79.5 (1.2)	86.1 (0.3)	57.3 (1.3)	71.5 (1.0)
MSMix	77.3 (−1.0)	86.1 (0.3)	59.4 (3.4)	71.6 (1.1)
DiSMix	**80.0** (1.7)	86.1 (0.3)	57.4 (1.4)	71.8 (1.3)
MDiSMix	77.9 (−0.4)	**86.3** (0.5)	**59.6** (3.6)	**72.0** (1.5)

**Table 4:** The results on VTAB-1k Natural. Results in bold indicate the best result.

Method	Caltech	CIFAR	Flowers	Pets
ERM	87.3 (±1.22)	73.3 (±2.28)	99.1 (±0.20)	87.3 (±1.27)
Mixup	88.2 (±1.03)	74.7 (±1.82)	98.9 (±0.33)	89.0 (±0.24)
Manifold	88.5 (±1.33)	69.7 (±2.98)	98.3 (±0.69)	85.8 (±2.22)
SMix	88.7 (±0.71)	74.5 (±1.88)	99.1 (±0.09)	88.4 (±0.80)
MSMix	88.3 (±1.66)	69.1 (±3.83)	98.3 (±0.34)	85.9 (±1.47)
DiSMix	89.0 (±0.77)	76.3 (±1.42)	99.0 (±0.20)	88.9 (±0.45)
MDiSMix	88.7 (±1.35)	70.8 (±3.51)	98.6 (±0.56)	86.7 (±1.30)

**Table 5:** The results on VTAB-1k Natural(2). Results in bold indicate the best result.

Method	Sun397	DTD	SVHN
ERM	40.7 (±1.22)	70.7 (±1.34)	89.5 (±1.12)
Mixup	41.3 (±0.71)	72.3 (±0.91)	90.5 (±0.49)
Manifold	36.4 (±3.75)	69.7 (±2.98)	91.5 (±0.72)
SMix	41.4 (±1.15)	72.0 (±1.29)	91.9 (±0.69)
MSMix	36.3 (±2.72)	70.7 (±1.16)	91.8 (±0.86)
DiSMix	41.7 (±0.84)	72.4 (±0.71)	92.1 (±0.39)
MDiSMix	36.9 (±2.98)	71.6 (±1.17)	91.9 (±0.52)

**Table 6:** Results on VTAB-1k Natural with SwinV2. Results in bold indicate the best result.

Method	Caltech	CIFAR	Flowers	Pets
ERM	88.0	76.5	99.5	91.8
Mixup	88.9	78.8	99.5	91.7
SMix	88.7	77.2	99.4	91.7
DiSMix	89.3	78.6	99.6	91.9

**Table 7:** Results on VTAB-1k Natural with SwinV2(2). Results in bold indicate the best result.

Method	Sun397	DTD	SVHN	Average
ERM	47.4	75.4	91.6	81.4
Mixup	48.6	75.1	93.1	82.2
SMix	48.5	75.0	93.7	82.0
DiSMix	49.0	76.0	93.5	82.6

**Table 8:** Increases in accuracy when changing the condition from α=1.0 to α=0.5.

Method	Caltech	CIFAR	Flowers	Pets
Mixup	0.0	+2.8	+0.0	+0.5
SMix	−0.2	+4.6	+0.4	+0.3
DiSMix	−0.5	+2.2	+0.1	−0.3

**Table 9:** Increases in accuracy when changing the condition from α=1.0 to α=0.5(2).

Method	Sun397	DTD	SVHN
Mixup	+1.1	+2.0	+0.4
SMix	+2.6	+1.5	+0.3
DiSMix	+2.0	+1.8	+0.4

## Data Availability

The data presented in this study are openly available in Visual Task Adaptation Benchmark (VTAB) at https://github.com/google-research/task_adaptation (accessed on 1 April 2025).

## Abbreviations

The following abbreviations are used in this manuscript:

ViT	Vision Transformer
DISMix	Dimensional Swap Mix

## Appendix A. VTAB-1k

VTAB-1k is a set of downstream tasks, consisting of the datasets shown in app (Table A1). The dataset is classified into 3 subgroups (Natural, Specialized, Structured) according to the category of the task. This set is the standard metric for evaluating parameter-efficient fine tuning. The training examples available for training comprise 800 pairs taken from the original dataset, and we aim to improve accuracy with a limited number of training samples compared to the original downstream task.

**Table A1:** List of datasets included in VTAB-1K.

Subgroup	Dataset	Classes	Test
	Caltech101	102	6084
	CIFAR-100	102	10,000
	Sun397	397	21,750
Natural	Flowers102	102	6149
	Pets	37	3669
	DTD	47	1880
	SVHN	10	26,032
Specialized	EuroSAT	10	5400
	Resisc45	45	6300
	PatchCamelyon	2	32,768
	Retinopathy	5	42,670
Structured	dSprites	16	73,728
	location/orientation		
	SmallNORB	18/9	12,150
	azimuth/elevation		
	Clevr	8/6	15,000
	count/distance		
	KITTI	4	711
	DMLab	6	227,351

## Appendix B. Base Learning Rate for VTAB-1k Structure

We found that the optimal learning rate for VTAB-1k Structured differs between tasks. Therefore, we performed a grid search of learning rates in the range of 1.6 × 10^−6^ to 1.6 × 10^−3^ for tasks classified as VTAB-1k Structured. The results are found in Table A2. The left grid search was performed only for the learning rate of ERM, and the learning rate that recorded the highest accuracy was set as the Base LR.

**Table A2:** Learning rate of VTAB-1k Structured.

Dataset		Learning Rate
dSprites	location	$8.0\times 10^{-4}$
	orientation	$1.3\times 10^{-5}$
SmallNORB	Azimuth	$1.0\times 10^{-4}$
	Elevation	$3.2\times 10^{-6}$
Clevr	count	$5.0\times 10^{-5}$
	distance	$1.0\times 10^{-4}$
KITTI		$1.3\times 10^{-5}$
DMLab		$5.0\times 10^{-4}$

## Appendix C. Each Result of VTAB-1k

Table A3–Table A5 present the results of VTAB-1k Structured. MDiSMix outperforms other methods on 3 out of 8 tasks. According to the VTAB-1k Structured, the preferable method is varied based on the task (Manifold and MSMix also record the highest scores on a task).

**Table A3:** The results on VTAB-1k Structured(1). Results in bold indicate the best result.

	dSprites		SmallNORB	
Method	Location	Orientation	Azimuth	Elevation
ERM	75.1	62.0	21.1	44.8
Mixup	76.8	67.2	22.2	38.6
Manifold	77.1	66.9	23.5	45.5
SMix	76.2	64.8	23.4	41.2
MSMix	75.6	64.4	22.8	46.3
DiSMix	75.6	65.2	23.1	39.3
MDiSMix	76.1	66.3	22.7	**47.8**

**Table A4:** The results on VTAB-1k Structured(2). Results in bold indicate the best result.

	Clevr			
Method	Count	Distance	Kitti	B
ERM	64.9	51.9	79.4	48.9
Mixup	54.6	58.5	77.9	53.5
Manifold	66.2	58.0	78.5	53.1
SMix	63.3	57.8	79.0	52.6
MSMix	**71.1**	58.7	**80.7**	53.5
DiSMix	66.5	58.3	78.5	52.9
MDiSMix	69.5	**60.3**	80.3	**53.8**

**Table A5:** The results on VTAB-1k Specialized. Results in bold indicate the best result.

Method	EuroSAT	Resisc45	Camelyon	Retinopathy
ERM	96.5	85.2	86.8	74.6
Mixup	96.9	87.8	85.4	74.5
Manifold	97.2	85.9	86.4	75.0
SMix	97.2	86.6	85.6	74.9
MSMix	97.0	86.1	86.0	75.0
DiSMix	97.3	86.8	85.4	74.8
MDiSMix	97.2	86.3	86.6	75.0
